# Microbiological Safety of Non-Food Products: What Can We Learn from the RAPEX Database?

**DOI:** 10.3390/ijerph16091599

**Published:** 2019-05-07

**Authors:** Szilvia Vincze, Sascha Al Dahouk, Ralf Dieckmann

**Affiliations:** Department of Biological Safety, German Federal Institute for Risk Assessment, 10589 Berlin, Germany; Sascha.Al-Dahouk@gmx.de (S.A.D.); ralf.dieckmann@bfr.bund.de (R.D.)

**Keywords:** microbial contamination, non-food products, RAPEX, rapid alert system of dangerous non-food products

## Abstract

For consumer protection across borders, the European Union has established the rapid alert system for dangerous non-food products (RAPEX), with the overarching goal of preventing or limiting the sale and use of non-food products that present a serious risk for the health and safety of consumers. In our study, we comprehensively analyzed RAPEX notifications associated with products posing a microbiological risk from 2005 through 2017. Additional information was retrieved from national laboratory reports. A total of 243 microbiologically harmful consumer products triggered notifications in 23 out of 31 participating countries. About half of the products were reported by Spain, Germany, and Italy. Notifications mainly included contaminated toys, cosmetics, and chemical products. Depending on the notifying country, measures taken to prevent the spread of dangerous products were predominantly ordered either by public authorities or economic operators. The interval between microbiological diagnosis and the date of RAPEX notifications considerably varied between RAPEX member states, ranging between a few days and 82 weeks. The nature and extent of RAPEX usage substantially differed among member states, calling for harmonization and optimization. Slight modifications to RAPEX could help to systematically record microbiological hazards, which may improve the assessment of potential health risks due to contaminated non-food products.

## 1. Introduction

Public health services dedicate a lot of time and effort to identifying and eradicating putative infection sources. In recent years, the awareness of the microbiological risks of non-food products has increased. Contaminated cosmetic products [1,2,3,4,5,6,7] as well as unsterile tattoo inks (reviewed in [8]) have been frequently reported as causative vectors of human infection. Globalized markets enable the quick spread of these harmful products within and between countries, which may lead to regional and supraregional outbreaks. 

For consumer protection across borders, the European Union (EU) has established the rapid alert system for dangerous non-food products (Rapid Exchange of Information System, or RAPEX) with the overarching goal of preventing or limiting the sale and use of non-food and non-pharmaceutical products that are presenting a serious risk for health and safety of consumers. Under Article 12 of the general product safety directive (2001/95/EC) RAPEX was established as a mandatory reporting system for all EU member states and the three European Economic Area (EEA) countries: Norway, Iceland, and Liechtenstein (RAPEX member states).

In 2009, the European Commission defined standards to harmonize the reporting system for non-food products (2010/15/EU). According to these specified guidelines, RAPEX member states (MS) have a legal obligation to inform the European Commission about hazardous products, provided that all of the following criteria are met (literally cited):

‒ The product is a consumer product;
‒ The product is subject to measures that prevent, restrict, or impose specific conditions on its possible marketing or use (“preventive and restrictive measures”);
‒ The product poses a serious risk to the health and safety of consumers;
‒ The serious risk has a cross-border effect.

Beside general regulations on product safety given in directive 2001/95/EC, legislative specifications on microbiologically hazardous products are available at the EU level for cosmetics (regulation EC No. 1223/2009) and toys (directive 2009/48/EC). For both product categories, maximum microbiological contamination levels are defined. In addition, specific pathogens are listed that must be absent from the product. 

Cosmetics are classified into two categories, based on the consumer group and application. Category 1 is comprised of products specifically intended for children under the age of three, and products applied in the eye area or on mucous membranes. Category 2 includes all other cosmetic products. Quantitative limits for the total viable count of aerobic mesophilic microorganisms are set at 10^2^ colony forming units (cfu)/g or ml in Category 1 products, and 10^3^ cfu/g or mL in Category 2 products. *Pseudomonas (P.) aeruginosa*, *Staphylococcus (S.) aureus*, and *Candida albicans* must be absent from a representative product sample of 1 g or mL (Categories 1 and 2) [9]. Furthermore, the resolution ResAP (2008) defines the basis for tattoo product safety [10], stating that tattoo and permanent make-up products should be sterile.

In toys, *S. aureus*, *P. aeruginosa*, *Candida albicans*, *E. coli*, or *Salmonella* spp. have to be absent from a representative product sample of 1 mL or g. Quantitative limits are set at ≤10^3^ cfu/g or mL for the total aerobic microbial count, and ≤100 cfu/g or mL for yeast, mould, and *Enterobacteriaceae* [11]. 

Based on these EU regulations, resolutions and guidelines of the European Expert Group, as well as complementary national legislation, RAPEX MS have a surveillance tool for microbiologically dangerous non-food products in place, and are consequently able to take countermeasures that prevent the spread of potentially hazardous products. Furthermore, the RAPEX platform enables quick exchange of information between RAPEX MS, and thus helps to prevent and restrict the supply of dangerous products across borders, which will finally increase consumer safety in Europe.

In our study, we comprehensively analyzed RAPEX notifications, published between 2005 and 2017, that are associated with products posing a microbiological risk for consumers, with the aim of highlighting the strengths of the platform and discussing development opportunities. 

## 2. Materials and Methods 

Publicly available data were extracted from the RAPEX platform [12] on 29 January 2018. The following inclusion criteria were applied: Years “2005–2017” and risk type “microbiological”. National laboratory reports on 222 notified products, provided by the German RAPEX contact point at the Federal Institute for Occupational Safety and Health, added information on the microorganisms involved and/or the specific finalization date of each report. Descriptive statistics were performed with IBM SPSS Statistics version 21 (IBM corp., Armonk, NY, USA).

## 3. Results

### 3.1. Product Categories and Countries Involved

**Figure 1:**
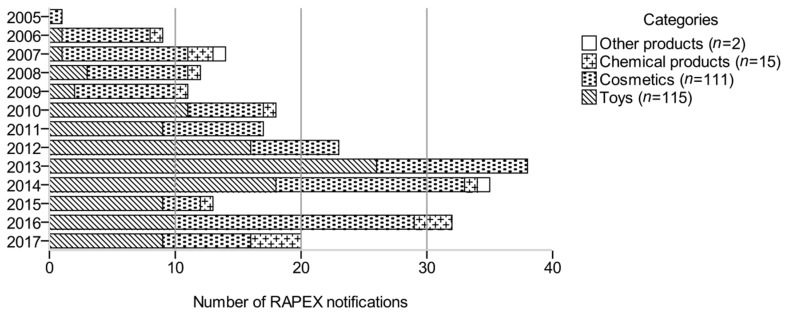
Rapid Exchange of Information System (RAPEX) notifications for products posing a microbiological risk, assigned to product categories for each year (2005–2017).

Between 2005 and 2017, 21,641 reports were published on the RAPEX platform, referring to 1 out of 20 risk types. A small fraction of these reports (1.1%; *n* = 243) dealt with microbiologically harmful consumer products. These notifications reported on products of various categories: toys (*n* = 115; 47.3%), cosmetics (*n* = 111; 45.7%), chemical products (*n* = 15; 6.2%), and other products (*n* = 2; 0.8%) (Figure 1). Affected toys were mainly associated with soap bubbles (*n* = 93). Cosmetics were comprised of products for children <3 years of age (*n* = 8), eye application (*n* = 13), use on mucosal membranes (*n* = 13), and other cosmetic products (*n* = 77). Chemical products predominantly included tattoo inks (*n* = 12). The category other products summarized a case of tattoo needles and one of water filters.

After a single report on microbiological risks in non-food products in 2005, the total number of notifications per year ranged between 9 and 38 during the study period. Cosmetics and toys were the most frequently affected product categories. Between 2006 and 2017, an average of 10 toys (ranging from 1 to 26), nine cosmetics (3–19), and one chemical product (0–4) were reported each year (Figure 1). 

Cosmetic products were often the subject of notifications in 2013 (*n* = 12), 2014 (*n* = 15), and 2016 (*n* = 19), whereas only three cosmetic products were reported in 2015. Toys were sporadically reported (1–3 products/year) between 2006 and 2009. In 2010, a sharp increase was observed (*n* = 11). Since then, the number of notified toys has not substantially decreased. However, a notification peak attracted attention in 2013, when alerts were published for 26 different toys due to microbial contamination. 

Twenty-three out of 31 countries participating in the RAPEX system submitted initial notifications, with frequencies ranging between 1 and 49 reports per country. About half of the microbiologically contaminated products (*n* = 130; 53.5%) were reported by only three countries, namely Spain (*n* = 49; 20.2%), Germany (*n* = 44; 18.1%), and Italy (*n* = 37; 15.2%). While Spain and Italy mainly reported contaminated toys, Germany predominantly reported cosmetic products (Table 1). A total of 158 products (65%) were imported from non-European countries, with China being the main producing country (*n* = 111; 45.7%). Eighty products (32.9%) originated from European countries, and 42 of them were reported by their country of origin. For five products, information on the source country was not available (Table 2). 

While some countries like Spain and Italy mainly reported products from non-European countries, other countries such as Germany equally reported products manufactured in Europe and in third countries (Table 3).

### 3.2. Microbiological Hazards

For 240 products, the microbiological risk was based on contamination. Either total aerobic microbial counts exceeded predefined limits, or specific potential pathogens were identified according to EU regulations for cosmetics and toys, as well as the resolution for tattoo products [9,10,11]. Tattoo needles and water filters were reported once in the study period, due to insufficient sterilization and reduced filtering capacity compared to the user manual, respectively. The actual reporting reason was missing for one tattoo ink. 

Exceeding limits of total aerobic microbial count (bacteria and fungi) were the main notification reason in the toys (93%; *n* = 107), cosmetics (89%; *n* = 99), and chemical products (86%; *n* = 12) categories. Additional information on the microorganisms responsible for contamination was available for about half of these products (toys: *n* = 49; cosmetics: *n* = 57; chemical products: *n* = 5). Identification of specific microorganisms [9,10,11] led to objection in 7% (*n* = 8), 11% *(n* = 12), and 14% (*n* = 2) of the products within the categories toys, cosmetics, and chemical products, respectively. 

**Figure 2:**
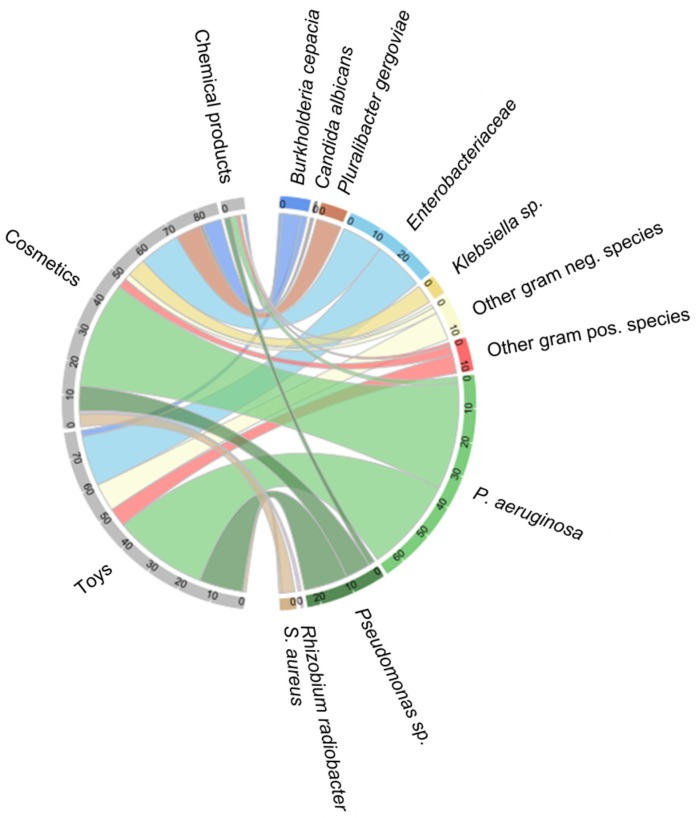
Microorganisms identified in the product categories toys, cosmetics, and chemicals. The arc lengths on the outer circle are proportional to the fractions of the product categories and number of microorganisms.

More detailed data on microbial characteristics were available for 133 products. Characterization provided information on family (*Enterobacteriaceae*), genus, or species, or was limited to Gram staining results. One to four different organisms per product were listed. In total, 171 microorganisms were categorized in 1 out of 11 subgroups, including *P. aeruginosa* (*n* = 66), other *Pseudomonas* spp. (*n* = 24), *Enterobacteriaceae* (*n* = 29), other Gram negative species (*n* = 12), other Gram positive species (*n* = 10), *Burkholderia (B.) cepacia* (*n* = 9), *Pluralibacter gergoviae* (formerly *Enterobacter gergoviae* [13]) (*n* = 8), *Klebsiella* spp. (*n* = 6), *S. aureus* (*n* = 5), *Rhizobium radiobacter* (*n* = 1), and *Candida albicans* (*n* = 1). Species distribution in toys, cosmetics, and chemical products showed similar patterns for frequently identified bacteria (*P. aeruginosa*, *Pseudomonas* sp., *Enterobacteriaceae*). *Pluralibacter gergoviae* and *Klebsiella* spp. were exclusively identified in cosmetic products (Figure 2). 

### 3.3. Types of Notifications and Measures Taken

Measures are either categorized as compulsory, if ordered by national authorities, or voluntary, if taken on the initiative of the producer, importer, or distributor of the dangerous product (economic operator). In case of microbiological objection, actions have included withdrawal of the product, sales ban, recall from the consumer, or rejection of import. Information was provided in 241 reports, including 146 (60%) compulsory and 95 (39%) voluntary measures. Comparison between the main reporting countries Spain, Germany, Italy, and France revealed differences in the involvement of actors responsible for notification. In Spain and Italy, measures were predominantly ordered by national authorities. In contrast, economic operators most frequently were the drivers for action in France and Germany (Table 3).

Measures initiated by economic operators were equally associated with products originating from Europe (*n* = 46) and non-European countries (*n* = 49). Products from Europe included those traded between EU and EEA member states (*n* = 18), as well as domestic products (*n* = 28). In contrast, the majority of measures ordered by public authorities were taken for products manufactured in non-European countries (*n* = 108). Reported products originating from Europe included those traded between EU and EEA member states (*n* = 19), as well as domestic products (*n* = 14). For five notifications reporting compulsory measures, the source country of the product was not available. From 2006 through 2017, the proportion of voluntary measures per year ranged between 28 and 48%, without a clear trend. There was only one outlier in 2007, when voluntary measures peaked with 71%. 

Measures taken by other European countries because of an initial RAPEX alert need to be notified to the RAPEX platform, and are registered as additional notification in association with the affected product. Such additional notifications were registered for 20% (*n* = 48) of the products concerned, including 22 cosmetics, 23 toys, and three chemical products. Thereby, the number of other reporting countries ranged between one and nine per product. Twenty-five countries additionally reported products on the basis of already published reports. These products were intended either for use by children (*n* = 25) or by adults (*n* = 23). Microbiological safety was mainly compromised due to high bacterial counts (*n* = 45). More details on the contaminating microorganisms were available for 19 products, including *P. aeruginosa* (*n* = 9), other *Pseudomonas* spp. (*n* = 8), *Pluralibacter gergoviae* (*n* = 6), *B. cepacia* (*n* = 3), *Enterobacteriaceae* (*n* = 2), and *S. aureus* (*n* = 1). Measures were either ordered by public authorities (*n* = 21) or directly taken by economic operators (*n* = 27). 

### 3.4. Interval between Microbiological Diagnosis and Date of RAPEX Notification

**Figure 3:**
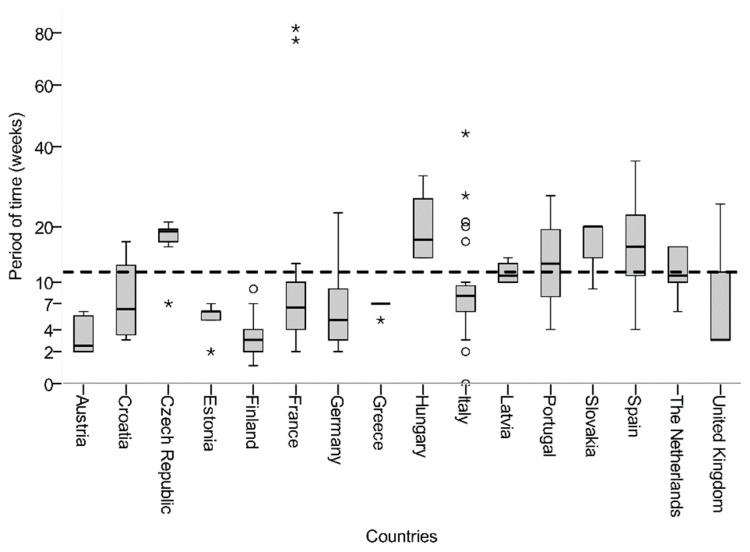
Boxplot graph presenting the time period between completed microbiological examination (national laboratory report) and publication date of the corresponding RAPEX notification, shown by country. The black dashed line represents the mean diagnostic delay of 11.6 weeks (standard deviation ± 10.4 weeks) for all reported products with available national laboratory reports (*n* = 209). The circles define outliers with values between 1.5 and 3 times the height of the boxes. Asterisks display extreme outliers, representing cases with values of more than three times the height of the boxes.

To evaluate process speed, the period between finalization of national laboratory reports and publication date of the corresponding RAPEX alert was determined. These data were available for 86% (*n* = 209) of the notifications. The average interval between finalization of the national laboratory report and published RAPEX notification was 11.6 ± 10.4 weeks (median: 8 weeks). The reporting delay varied substantially, ranging between a few days and 82 weeks. Mean reporting delays considerably differed between notifying countries (Figure 3). Countries with fewer than three complete data sets were excluded from the comparative analysis (Belgium, Bulgaria, Denmark, Ireland, Lithuania, Norway, and Poland). 

## 4. Discussion

### 4.1. Identified Microbiological Hazards

Analysis of RAPEX alerts revealed notifications in the category microbiological risk on a regular basis. Cosmetics and soap bubble toys were mainly affected. This is in accordance with other investigations of the RAPEX system. For cosmetics, previous studies identified microbiological risks as the second leading cause of notifications in this product category (Germany: 2005–2017 [14], all EU member states: 2008–2014 [15]).

While reporting frequencies remained relatively stable for cosmetics and chemical products during the study period, a sharp increase of notifications on contaminated toys was observed in 2013, followed by a continuous decrease in the following years, until the number of reports reached a level similar to that of the period 2009–2011. The tremendous change in the number of reports for a limited time suggests an increased awareness of microbiological risks in toys. Our literature search on this topic revealed three cases of septicemia in children after playing with contaminated soap bubble toys in 2011 [16]. The case report in 2011 might have been the kickoff for the increasing trend of RAPEX alerts related to soap bubble toys, indicating an enhanced risk perception. Interestingly, the altered reporting behavior mainly occurred in Italy, where the primary outbreak was described, and in Spain. Twenty-three and 36 soap bubble toys were reported from Italy and Spain, respectively, during the evaluation period (2005–2017). Other European countries showed low to moderate reporting levels, ranging between 0 and 10 products per country. Possible explanations for the decreased number of notifications about contaminated soap bubble toys starting in 2015 might be a lower interest of public health services four years after the outbreak, which might have resulted in reduced monitoring or the implementation of stricter hygiene standards by manufacturers, leading to a lower number of microbiologically contaminated soap bubble toys on the market. As no data are available on the total number of investigated soap bubble products, conclusions remain speculative.

The main microbiological hazard identified was a total aerobic microbial count that exceeded the limits determined by EU regulation for toys and cosmetics [9,11]. In the category chemical products, tattoo inks were mainly reported because they were non-sterile. The microorganisms predominantly identified in consumer products are ubiquitous opportunistic pathogens that usually pose little risk to healthy adults, but which can cause serious infections in immunocompromised people. *Pseudomonas* spp. and especially *P. aeruginosa* were the microorganisms most frequently found in cosmetics and toys. This is in accordance with previous studies, which identified *Pseudomonas* spp. as common contaminants of cosmetics [7,14,15,17,18,19,20]. *P. aeruginosa* may cause corneal ulcer or sepsis as a result of using contaminated cosmetic products [7,18]. To date, data on the occurrence of microorganisms in toys are rare and patchy. The aforementioned case report describing an infectious outbreak among children who played with contaminated soap bubble toys identified *P. aeruginosa* as the causative pathogen [16]. *Enterobacteriaceae* were the second largest group identified in cosmetics and toys. *Klebsiella* spp. were regularly isolated from cosmetic products and pharmaceutical hand soaps [20,21,22,23,24]. *Klebsiella* may also cause outbreaks, which have been described as a consequence of the use of contaminated hand cream [24]. According to the RAPEX dataset, the ubiquitous microorganism *Pluralibacter gergoviae* was exclusively isolated from cosmetics, as previously described [19,20,25]. Molecular analysis revealed an intrinsic resistance mechanism of *Pluralibacter gergoviae* to parabens and other biocides that are frequently used as preservatives in cosmetics [26]. Knowledge on the pathogenic potential of this opportunistic pathogen is limited, with only a few human infections reported, including abdominal abscess as well as sepsis [27,28]. In these cases, isolates showed multidrug resistance, which might impact therapeutic options. *B. cepacia* was identified in all product categories. Pathogenicity of *B. cepacia* is usually restricted to immunocompromised individuals. In this context, *B. cepacia*-contaminated mouthwash was responsible for life-threatening infections in hospitalized patients [1,2,22,29]. Because of intrinsic resistance to the vast majority of clinically relevant antimicrobials, the occurrence of *B. cepacia* in consumer products is alarming [30]. The opportunistic pathogen *S. aureus* was identified in cosmetics and toys. Both intrinsic and extrinsic contaminations of cosmetics have been described [17,31,32]. However, a direct link between human infections and *S. aureus*-contaminated non-food products has not been reported yet.

The reporting frequency of bacterial species in RAPEX notifications needs to be interpreted with caution, since testing for the presence of specific bacterial species, including *P. aeruginosa* and *S. aureus*, is mandatory, while other microorganisms are often summarized as aerobic microbiological contamination. Thus, it is currently not possible to identify which microorganisms are the main contaminants of non-food products. For this purpose, the bacterial species should be provided in each notification.

### 4.2. Current Application of RAPEX and Limitations of the System

The rapid alert system for dangerous non-food products was implemented to prevent or restrict the spread of hazardous products within and between RAPEX MS. Different prerequisites are necessary to achieve this goal, including:

1. Active participation of all RAPEX MS,
2. Fast exchange of information between EU and EEA member states,
3. Early identification of microbiologically hazardous products.

#### 4.2.1. Active Participation of all RAPEX Member States 

The RAPEX system enables EU and EEA member states to actively participate in two ways. First, they have the obligation to report products posing a serious risk for the consumer. Second, they must additionally report follow-up actions taken in reaction to initial notifications (additional notifications).

The number of submitted initial alerts varied substantially between participating countries, ranging from absence of initial notifications to 49 alerts, with Spain, Germany, and Italy as the main reporting countries. Various factors may influence the reporting behavior of a country, including size of sales market and import volumes. The three main reporting countries have comparably large markets. However, this is also true for other countries like France and the United Kingdom that had lower reporting frequencies, which indicates that additional factors may drive notification behavior. Historical differences in risk perception and risk assessment, for example, might influence the awareness of a population. Furthermore, the number of routine samples tested can simply increase the number of notifications.

Additional notifications were published for 20% of the microbiologically harmful products. Comparison of alert characteristics did not reveal any association between product-specific properties and reactions. However, country-specific analysis identified variability in the application of the RAPEX platform. While some countries registered both initial notifications and reactions, other countries used the RAPEX platform only for one of the two purposes. Slovenia was one of the countries with a high number of additional notifications, although no initial reports were registered in the category microbiological risk. In contrast, Italy—one of the major reporting countries—did not report reactions based on initial alerts.

The large variability in the reporting frequency of reactions ranging between 0 and 11 additional notifications during the investigation period indicates that the use of RAPEX is still not harmonized. A systematic evaluation of initial notifications may help RAPEX MS to minimize the presence of microbiologically harmful products on the market in a resource-saving manner, and thus help to increase consumer safety.

#### 4.2.2. Fast Exchange of Information between European Union and European Economic Area Member States

Rapid exchange of information on identified harmful products is necessary to efficiently protect consumer health. The reporting delay in the RAPEX system after the finalization of laboratory reports on the national level varied widely, ranging between 1 week and 82 weeks (mean reporting delay ± standard deviation = 11.6 ± 10.4 weeks). The longer the delay, the wider contaminated products may be distributed. To avoid the spread of health risks in the EU, the RAPEX guidelines define rules on the timing of notifications for the member states and the Commission. Deadlines are set starting with the implementation of measures in the reporting country. Unfortunately, the dates when measures were taken are not notified via RAPEX. Thus, it is impossible to clarify whether the reporting delay occurred before or after measures were implemented. Differences in the national notification systems and case-specific features might explain the large range in the reporting delay. However, variation within individual countries indicates room for improvement independent of national reporting systems.

#### 4.2.3. Early Identification of Microbiologically Hazardous Products 

Ideally, potential health risks should be identified in the manufacturing country so that measures can be taken at the earliest possible time in the product’s lifecycle, in order to minimize the distribution of dangerous goods. The overall proportion of domestic notifications in RAPEX MS was moderate (52.5% of all products manufactured in Europe). However, substantial differences were observed between notifying countries. While some countries, like Spain and Italy, did not report any or notified only a few domestic products, the proportion of domestic notifications was considerably higher in other member states, including Germany and France. Hence, there is a good chance of improving the RAPEX system.

In total, 65% of the reported products originated from non-European countries, with China as one of the main countries of origin (45.7%). This finding is in accordance with the general trend of reported products from all risk categories published in the annual RAPEX report for 2016 [33]. Due to the high prevalence of affected Chinese products, a close cooperation system (RAPEX-China) was established in 2006 to reduce the presence of unsafe Chinese products on the European market. In this context, RAPEX provides a module that flags reported Chinese products and provides access for Chinese authorities, allowing them to increase awareness of product safety rules in Europe among Chinese manufacturers, exporters, and businesses. This close collaboration has resulted in a gradual decline of RAPEX notifications for Chinese products in all risk categories since 2013 [33].

Strengthening the responsibility of economic operators could help to identify harmful products at an early stage. Economic operators have to ensure the safety of their products. Therefore, they need to implement and regularly evaluate internal quality controls. After identification of a putative harmful product that is already placed on the market, the responsible economic operator has to inform national authorities to initiate measures that prevent further release of the product for sale. Thus, the RAPEX platform can be used to inform participating EU and EEA member states about harmful products that have been identified during systematic routine quality controls by economic operators. According to the notifications under study that reported microbiological risks, compulsory and voluntary measures were equally often taken. However, the notification basis of the individual RAPEX MS displayed a very heterogeneous pattern, with measures either predominantly taken by public authorities (in Spain and Italy) or initiated by economic operators (in Germany and France). Only few countries like The Netherlands showed relatively balanced numbers of compulsory and voluntary measures.

While identification of hazardous products by economic operators is based on internal quality controls reflecting compliance, official controls are implemented for surveillance of previously defined quality standards. Thus, routine sampling identifies harmful products only by chance. The detection rate of harmful products depends on numerous factors, including sample size and sample diversity. In Germany, for example, controls are conducted by veterinary and food control authorities, and can be either based on the federal control plan, which is released yearly by the Federal Office of Consumer Protection and Food Safety, or by other coordinated programs at the state level. A total of 0.5 non-food products per 1000 inhabitants each year (roughly 40,000 products, including tobacco products, cosmetics, and commodities) need to be investigated. The overarching goal of the control plan is to harmonize the controls for food and non-food products in the federal states. Furthermore, the control plan defines risk-based inspection priorities. In 2017, the inspection focused on isothiazolinones in cosmetics, the release of boron and barium from knead- and slime-based toys, and the presence of hydroquinone and hydroquinone methylether in nail gels in the categories commodities and cosmetic products [34]. The targeted but limited evaluation of consumer products may explain the low number of notifications based on compulsory measures in Germany.

## 5. Conclusions

Conclusions beyond the current scope of RAPEX include the question: can the platform help to identify microbiological hazards of non-food products?

In general, information is lacking on the prevalence of opportunistic infections caused by microbiologically contaminated non-food products, which hampers sound risk assessment. Nonetheless, various studies report mild to severe infections due to microbes in tattoo inks, toys, and cosmetic products [1,2,3,4,5,6,7,8]. The overall frequency of these events remains largely unknown, since sources of opportunistic infections are rarely traced back. This is why the question may arise whether the hitherto published cases only reflect the tip of the iceberg. RAPEX could partly help to answer this question. However, the notification system does not record all dangerous non-food products on the market, since it is restricted to those with cross-border effects. Nevertheless, the system may help to identify and analyze risk trends, as observed for contaminated soap bubble toys. Unfortunately, current notifications do not necessarily reflect scientific knowledge on microbiological contamination rates in consumer goods, a phenomenon that can be exemplarily observed for tattoo inks. While several studies reported high contamination rates in sealed tattoo inks and described the putative relevance for public health [8,35,36,37], this hazard seems to be highly underreported by the RAPEX notifications under study. Various factors could be responsible for the reporting gap, including sampling bias, use of different methods to identify microbiological risks, and discrepancies in the way of notification between countries.

The sampling bias could be reduced in two ways. First, the responsibility of economic operators to report microbiological contamination should be strengthened, since they detect putatively harmful products at a very early stage during internal controls. In the past, this strategy has been successfully applied in the European Rapid Alert System for Food and Feed (RASFF) [38]. Second, EU-wide control plans for non-food products could be implemented to harmonize sampling procedures. However, the huge diversity of non-food products makes systematic screening a challenge. Thus, risk-based sampling should be carried out in consideration of up-to-date scientific knowledge. Defining individual product groups that are officially controlled in EU and EEA member states on a regular basis might add value to the RAPEX system. With this approach, information on the prevalence of microbiological contamination in relevant non-food products can be gathered systematically. Such a database may help to annually adjust the focus of an EU-wide control plan.

The detection of microbes in non-food products should be based on EU-wide harmonized laboratory methods, like those established for food and feed in official reference laboratories. In the non-food sector, efforts have been made to implement EU guidelines for analytical testing of cosmetics, published by the Joint Research Centre [39]. Unfortunately, they do not include harmonized procedures for microbiological testing.

Moreover, it might be worth it to foster consumer engagement within the reporting system, which has been recommended by European Food Safety Authority (EFSA) in its 2020 strategy report [40]. Currently, interested consumers can retrieve information on notified products from the RAPEX platform, but are not able to report potentially hazardous products, although health risks are often identified when products are applied. This approach has been successfully established in the United States, where individual consumers are able to report unsafe products (https://www.saferproducts.gov) [41]. The collected data are used by the United States Consumer Product Safety Commission to identify and monitor health hazards.

In summary, the overarching goal of RAPEX is to enhance consumer safety by the quick exchange of information. Our analysis showed that the notification system is widely used. However, the nature and extent of its use differs considerably among EU and EEA member states, calling for harmonization and optimization. Slight modifications to the RAPEX notification system could help to systematically record microbiological hazards, which may improve the assessment of potential health risks due to microbiologically contaminated non-food products.

## Figures and Tables

**Table 1:** Number of initial notifications by product category for the three main reporting countries (2005–2017).

Alert Submitting Country	Product Category *n*				Total Number of Notifications (*n*)
	Toys	Cosmetics	Chemical Products	Other Products	
Germany	5	35	4	0	44
Italy	29	0	7	1	37
Spain	41	7	1	0	49

**Table 2:** Number of initial notifications and reactions for products posing a microbiological risk (2005–2017) listed for each country. The manufacturing country named in the initial notification is given (domestic, other European Union (EU) and European Economic Area (EEA) member states (MS), China, and other non-EU countries).

Alert Submitting Country	Total Number of Initial Notifications *n* (%)	Initial Notifications per Country of Origin *n*					
		Domestic Notification	Other European Country	China	Other Non-European Country	Unknown	Total Number of Reactions *n* (%)
Spain	49 (20.2)	4	1	34	5	5	5 (5.3)
Germany	44 (18.1)	14	11	6	13		3 (3.2)
Italy	37 (15.2)		1	32	4		
France	20 (8.2)	5	1	9	5		11 (11.8)
The Netherlands	13 (5.3)	3	2	5	3		4 (4.3)
Finland	9 (3.7)		1	6	2		5 (5.3)
Slovakia	9 (3.7)		6		3		3 (3.2)
Austria	8 (3.3)	3	4	1			9 (9.7)
Czech Republic	7 (2.9)	2	2	1	2		1 (1.1)
Hungary	7 (2.9)		1	6			2 (2.2)
United Kingdom	7 (2.9)	3		2	2		2 (2.2)
Estonia	6 (2.5)	1	1		4		
Greece	5 (2.1)	5					7 (7.5)
Croatia	4 (1.6)		1	2	1		2 (2.2)
Latvia	4 (1.6)			3	1		1 (1.1)
Ireland	3 (1.2)		1	1	1		6 (6.4)
Portugal	3 (1.2)		2		1		5 (5.3)
Lithuania	2 (1.2)			2			1 (1.1)
Poland	2 (1.2)		1	1			
Belgium	1 (0.4)	1					
Bulgaria	1 (0.4)	1					
Denmark	1 (0.4)		1				5 (5.3)
Norway	1 (0.4)		1				1 (1.1)
Slovenia							9 (9.7)
Estonia							4 (4.3)
Iceland							2 (2.2)
Sweden							2 (2.2)
Republic of Cyprus							1 (1.1)
Luxembourg							1 (1.1)
Romania							1 (1.1)
Liechtenstein							

**Table 3:** Number of compulsory and voluntary measures per reporting country (2005–2017).

Alert Submitting Country	Compulsory Measures (*n*)	Voluntary Measures (*n*)	Unknown (*n*)	Total Number of Initial Notifications (*n*)
Spain	44	5		49
Germany	3	40	1	44
Italy	34	2	1	37
France	3	17		20
The Netherlands	8	5		13
Finland	9			9
Slovakia	7	2		9
Austria	2	6		8
Czech Republic	7			7
Hungary	7			7
United Kingdom		7		7
Estonia	3	3		6
Greece	5			5
Croatia	4			4
Latvia	2	2		4
Ireland	1	2		3
Portugal	3			3
Lithuania	2			2
Poland	1	1		2
Belgium		1		1
Bulgaria	1			1
Denmark		1		1
Norway		1		1
